# The isoenzyme pattern of LDH does not play a physiological role; except perhaps during fast transitions in energy metabolism

**DOI:** 10.18632/aging.100329

**Published:** 2011-05-12

**Authors:** Bjørn Quistorff, Niels Grunnet

**Affiliations:** ^1^ Dept. of Biomedical Sciences, The Faculty of Health Sciences, The University of Copenhagen, Copenhagen, 2200 Denmark

## Introduction

A paper describing increased brain lactate concentration with age recently caught our attension [1]. The proposed explanation for the increased brain lactate with age was a shift in the brain lactate dehydrogenase (LDH) isoenzyme pattern, which also occurred with age. While the observations are highly interesting, we found the explanation unlikely under steady state conditions, since LDH is regarded a near-equilibrium reaction and since the equilibrium constant of all the LDH isoenzymes, of course, are the same [2]. We therefore decided to evaluate the question further, including a brief historical review, since a quick examination of common textbooks of biochemistry on the LDH isoenzyme question suggests that it is in fact common place to confer significant physiological importance to the different kinetic properties of the isoforms of LDH [3-9]

## Historical

The concept of isoenzymes evolved in the late 1950's and early 1960's. In case of LDH it became clear that five isoenzymes numbered 1-5 could be distinguished according to electrophoretic mobility [10] and that there was a tissue specific distribution of these isoenzymes [10, 11]. The various isoenzymes appeared to be tetramers of two different subunits, M and H. Isoenzyme 5 is composed of M-subunits only and is the predominant form in skeletal muscle and isoenzyme 1 is composed of H-subunits only and is found primarily in heart tissue. Isoenzyme 2-4 contains both subunits. Later studies showed that two proteins M and H are the products of the genes *Ldh-A* and *Ldh-B*, respectively, and the LDH enzyme presents with the following five isoenzymes: M_4_, MH_3_, M_2_H_2_, M_3_H and H_4_.

In the 1960's it was furthermore demonstrated that the LDH isoenzymes showed different kinetic properties with respect to substrate affinity and inhibition, where the M-dominated forms have a 3.5 – 7 times higher K_m_ value for pyruvate than H-dominated. (Table 1). The H_4_ isoenzyme is furthermore sensitive to substrate inhibition by pyruvate at concentrations above ~0.2 mM, whereas the M_4_ isoenzyme appear little affected by pyruvate concentrations as high as 5 mM [12-14]. Also, the H_4_ isoenzyme exhibits substrate inhibition by lactate above 20-40 mM, whereas the M_4_ isoenzyme is much less inhibited by high lactate concentrations [14]. The structural basis for the differences in kinetic properties was recently reported [15]. The large majority of measurements of the mentioned kinetic parameters have been carried out at 20 or 25 °C. In this context it should be noted that K_m_-values for pyruvate increase with temperature [13, 16] and are doubled at 37 °C compared to 25 °C, and that substrate inhibition by pyruvate may be less pronounced at 37 °C than at 20-25 °C, especially for the heart enzyme [13, 17] although there is disagreement regarding pyruvate inhibition [16]. Thus, there may be only small differences, if any, in kinetic constants among the LDH isoforms at physiological temperature.

**Table 1.** Kinetic properties of the LDH isoenzymes of different species. K_m_ values are in mM and V_max_ given as U/g wet wt.

**K_m_. Pyruvate as substrate:**			
	H_4_ or heart M_4_ or muscle		Ref.
	K_m_	K_m_	
Rabbit	0.10	0.35	[14]
Rat		0.17-0.27	[27]
Bovine	0.14	1	[30]
Chicken	0.09	3.2	[30]
**K_m_. Lactate as substrate:**
	H_4_ or heart M_4_ or muscle		
	K_m_	K_m_	
Rabbit	9.34	23.0	[14]
Rat		15-17	[27]
Bovine	9	25	[30]
Chicken	7	40	[30]
**V_max_. Pyruvate as substrate:**			
V_max_ values in U/g wet wt.			
Species Tissue	V_max_	Ref.	
Human*	Brain	46	[31]
Human*	m. vastus	175	[31]
Rabbit*	m. soleus	167	[31]
Rabbit*	m. tibialis anterior	1458	[31]
Dog*	Heart	125	[31]
Rat	m. plantaris	740-889	[27]
Rat*	Liver	83	[31]

* Assuming 3.5 g wet wt/g dry wt.

Historically, the tissue characteristic LDH isoenzymes distribution led to hypotheses of possible functional implications. This concept was pioneered by Kaplans group in particular [18, 19], where it was stated: *“The susceptibility of heart lactic dehydrogenase (H subunits) to inhibition by pyruvate is compatible with adaption of the heart to aerobic metabolism of pyruvate and NADH. Skeletal muscle enzyme (M subunits), on the other hand, resists inhibition by pyruvate, and enables the tissue to derive energy from anaerobic pathways when oxygen is limited and pyruvate accumulates”* [18]. This somewhat uncritical interpretation of the biochemical function of LDH isoenzymes seems to have been transmitted through most textbooks since, as mentioned above (e.g. 3-9).

## Analysis of the physiological role of LDH isoenzymes

Below we discuss the possible physiological roles, if any, of the LDH isoenzymes with brain and skeletal muscle as examples.

The H-dominated isoenzymes have a lower K_m_ value for pyruvate and lactate than M-dominated forms and are inhibited by pyruvate concentrations in the physiological range (Table 1). As mentioned above this is often interpreted to cause LDH isoenzymes composed primarily of M-subunits to preferentially catalyze the reduction of pyruvate→ lactate and conversely, that isoenzymes composed primarily of H-subunits predominantly should catalyze oxidation of lactate to pyruvate [1, 3-9] However, in spite of changed kinetic constants the equilibrium constant, K_eq_, is the same for all isoenzymes since it is the same chemical reaction being catalyzed. This is stated in the Haldane *Equation*, relating K_eq_ with the kinetic constants of the forward and reverse reaction:

$${\text{K}}_{\text{eq}}=\left({\text{V}}_{\max\text{ f}}{\text{K}}_{\text{mp}}\right)/\left({\text{V}}_{\max\text{ r}}{\text{K}}_{\text{ms}}\right)$$

Furthermore, the total LDH activity is high in most tissues compared to metabolic flux (see Table 1) and the reaction therefore likely to be close to equilibrium under steady state conditions.

Consequently, it would seem unlikely that the LDH isoenzyme pattern in itself could influence the tissue lactate concentration. This conclusion was, in fact, also reached in a recent study performing mathematical modeling of the LDH reaction, concluding that the decisive parameter was the total LDH activity and not the isoenzyme pattern [20].

The situation may, however, be quite different during metabolic transitions in energy metabolism where in particular the glycolytic flux can undergo large rapid changes. We propose that under such conditions there may be significant physiological effect of the isoenzyme pattern of a given tissue.

In brain under normal physiological conditions it seems that the intracellular lactate concentration is significantly higher (2-4 mM) than in the arterial blood [21]. This implies that the LDH reaction is in fact, not a dead-end reaction as is normally assumed, since there will be a net concentration gradient mediated flux in the reaction. In the brain this is indicated by a small but statistically significant net output of lactate at rest [22, 23]. Theoretically, this could affect steady state lactate concentration as a result of a changed LDH isoenzyme pattern. However, with a high total activity of LDH this is likely to be a minor effect [20]. A different situation might apply during the initial phase of brain activation (e.g. as occurring when initiating intense mental or physical exercise), where there may be a burst of anaerobic metabolism in the brain [24, 25] and therefore presumably a sharp increase in the pyruvate concentration. In that case the kinetics of the intracellular lactate concentration could be affected by the LDH isoenzyme pattern. But again, this applies only during the non-steady state situation of the brain activation.

Such non-equlibrium effects on the LDH reaction should be even more visible in skeletal muscle upon the transition from rest to work. Here the glycolytic flux can easily change by a factor of 50 [26] and an active involvement of the LDH reaction is suggested by observed training effects on the kinetics of the LDH reaction [27]. Under these conditions the LDH reaction is likely to be temporarily far from equilibrium and the kinetic effects of the pyruvate concentration on the LDH flux could be large.

It is under such conditions that the prevalent high LDH activity becomes metabolically important by providing an efficient way of buffering the pyruvate concentration excursions and indeed supplying the necessary NADH to uphold the glycolytic flux and the resulting ATP production. In that context, it is interesting to consider what purpose the rather low K_i_ for pyruvate of the H-form might serve, since it would tend to slow down the “buffering” by the LDH reaction of the pyruvate concentration. One suggestion could be that it would keep the pyruvate high for a longer period of time in the initial phase of glycolytic flux acceleration would therefore favor a subsequent acceleration of the pyruvate dehydrogenase (PDH) pathway. But this suggestion does not seem to be supported by the low K_m_ of PDH for pyruvate, which is about an order of magnitude lower than even normal resting pyruvate concentrations [28].

Apart from PDH, there are however, several other reactions connecting pyruvate to the the intermediary metabolism. For the alanine amino-transferase (ALAT) reaction, a high pyruvate concentration would favor glutamate conversion to α-ketoglutarate and consequently an increase in the TCA intermediary pool, which would seem useful under conditions of an eminent TCA acceleration and could therefore be considered a feed forward mechanism. The same effect would result by way of the pyruvate carboxylase reaction.

Therefore, it is not unlikely that the isoenzyme pattern of LDH does after all play an important physiological role under non-steady state conditions, and a quantitative evaluation through mathematical modeling of the above suggestions are needed.

In the context of the present evaluation of the LDH reaction it is noteworthy that the beta cells of the pancreatic islets does not express *Ldh-A* or the lactate and pyruvate transporter *Mct1* [29]. This is likely to be linked with the special function of the glycolytic flux of these cells as a sensor mechanism of the glucose concentration. i.e., without LDH activity, and therefore no lactate buffering on the pyruvate concentration, the magnitude of the glycolytic flux may be directly transmitted to TCA flux and ATP formation and subsequent insulin release. If LDH was present in high amounts or if pyruvate or lactate could be taken up in the beta cells, a bout of intense physical exercise, which results in very significant increase in systemic lactate, would transmit a pyruvate signal to the beta cell mitochondria and therefore potentially cause an irrelevant insulin secretion.

## Conclusion

All in all we find that the LDH isoenzyme pattern most probably is without effect on the intracellular lactate concentration and the explanation advanced by [1], is probably incorrect since their experiments on aging effects reflect steady state conditions. However, during fast metabolic transitions involving significant changes in the pyruvate concentration, primarily resulting from major changes in glycolytic rate, the LDH isoenzyme pattern may well play an important role in the compounded metabolic response to altered energy metabolism. However, this latter part of the conclusion depends strongly on resolving the issue as to whether the reported K_m_ differences for pyruvate between LDH isoenzymes are actually present at 37° C [13, 16, 17].
